# Exploring sexual and reproductive health needs, barriers, and coping strategies of internally displaced women of reproductive ages in north-central Nigeria: A qualitative analysis

**DOI:** 10.1371/journal.pone.0309317

**Published:** 2024-12-06

**Authors:** Kwala Adline Okorafor, Joseph Okeibunor, Funmilola Folasade Oyinlola, Leopold Ouedraogo, Femi Rufus Tinuola

**Affiliations:** 1 Texila American University, Georgetown, Guyana; 2 World Health Organization, Regional Office for Africa, Brazzaville, Congo; 3 Department of Demography and Social Statistics, Faculty of Social Sciences, Obafemi Awolowo University, Ile-Ife, Nigeria; Kalahandi University, INDIA

## Abstract

**Context:**

Women and girls form a substantial proportion of the population of internally displaced people (IDP) in Nigeria, these vulnerable populations are at risk of sexual and reproductive health (SRH) rights violations and greater risk of unsafe abortion and high maternal deaths. IDP women’s living conditions are often precarious, exposing them to health risks, challenges are often faced due to lack of finance and other related factors to access health care services leading to them improvising health care services which is considered dangerous to their health. There is a gap in the study regarding the SRH needs of the IDP women and the alternative they opt for in meeting their sexual needs.

**Objective:**

This study explores the SRH needs, barriers and coping strategies of women of reproductive age in North Central IDP camps.

**Data and methods:**

A qualitative study was conducted between January 8^th^ and February 28^th^, 2024, in 4 internally displaced camps in Abuja and Benue among women of reproductive age. Using a well-structured interview guide for an in-depth interview, 14 respondents were purposively selected for the study. The women were interviewed to provide insight into the SRH needs, coping strategies and barriers to utilizing SRH services in the selected camps. Data analysis was conducted using Nvivo version 11.

**Results:**

The study revealed the SRH needs for specific family planning methods, free SRH services and antenatal facilities in the camp and the need for toilets. Without the SRH services women cope using herbs and concoctions, they also patronize traditional birth attendants and some of the barriers include finance, husbands’ approval and distance to health facilities.

**Conclusion:**

In conclusion, women in the IDP camp have SRH needs that need to be met as their coping strategies are not adequate but could complicate their sexual health. It therefore recommended the government should provide adequate medical personnel in the camp for easy access to SRH services.

## Background

Internally displaced people (IDPs) refer to individuals who have been forced out of their place of permanent residence but remain within a state border recognized by international law [1]. Displacement in societies occurs due to war, natural disasters, and the unfavourable effects of climate change, often leading to human rights abuses and causing displacement in various settings [1, 2]. Displacement in Nigeria is primarily due to insurrection, terrorism, and flooding, leading to people escaping their homes, belongings, and employment places [2].

Globally, 82 million individuals have been forced to flee due to political unrest and violence, with over half of them under 18 [3, 4]. Over the past decade, there has been a significant rise in IDPs, reaching 51 million, with one-third of them residing in Africa [5, 6]. Humanitarian crises, including forced relocation and unstable environments, heighten the vulnerability of sexual and reproductive health (SRH), particularly for women and girls [7, 8]. Approximately 3 million Nigerians (as of November 2021) had been forced from their homes due to violence from the Boko Haram terrorist group, other armed groups, and conflicts between farmers and herders, mostly in parts of the northeast region and the Middle Belt of the country, but also increasingly in North-West Nigeria [9].

Banditry surges in northcentral and northwestern states in recent years have caused a new category of displacement, mostly as a result of drought and rising youth unemployment [10]. In 2020, the National Emergency Management Agency reported 375 IDP camps across Nigeria, with 31 located in the north-central states of Niger, Nasarawa, and the Federal Capital Territory [11].

Conflicts and forced migration burden healthcare systems, limiting access to essential sexual and reproductive health (SRH) treatments. According to the United Nations High Commissioner for Refugees data, 2.3 million girls and 1.6 million women in Northeastern Nigeria require humanitarian assistance. Women and children in IDP situations face health issues and are particularly vulnerable to sociocultural, economic, and SRH challenges, which include sexual and gender-based violence (GBV) during and after relocation, increased risks of unsafe abortions, sexual assaults, early and forced marriages, and sexual exploitation among others [7, 8, 12–14]. In Northern Nigeria, there are 709 maternal fatalities for every 100,000 live births, which is much higher than the 20 in affluent countries. Unsafe abortion is one of the main causes of maternal mortality in Nigeria, and its prevalence has increased in Northeastern Nigeria as a result of the Boko Haram insurgency [15]. Sexual risk-taking and irregular intake of contraceptives are linked to mental health issues, which can result in risky abortions, STIs, and unintended pregnancies [16]. The ongoing insecurity in the IDP camps and the regular attacks on IDPs who go in search of jobs outside of it contribute to the continuation of these traumas [16]. In IDP situations, violence and abuse of alcohol and drugs are prevalent [16], which has detrimental effects on young women and girls who are already at risk.

According to Chiabuotu et al. (2021), reported that men concealed their failures and incapacity to cope with the difficulties of displacement by being physically aggressive, particularly against their spouses, heavy alcohol consumption, sexual promiscuity, as well as taking on multiple marriages in their research on married persons living in an IDP camp in northeast Nigeria [15]. Hunger and poverty have an impact on the sexual and reproductive health of girls and young women due to the possibility of trading sex for food or cash. A study by [16], interviewed participants talked about how their inability to afford food or other necessities prompted them to trade sex for food and money and how parents push their teenage daughters into forced or young marriages or similar situations, putting them at risk for STIs, premature pregnancy, and gender-based violence [16].

Due to the stigma associated with their age and marital status, younger single women and girls were reluctant to seek pregnancy assistance in camp clinics [16]. In other camps for IDPs in Africa, it has been shown that girls and young women underutilize health care because of feelings of humiliation, embarrassment, fear of reprisals, and social discrimination [17]. Since the camp only provided post-abortion care and did not offer induced abortion services, young women were forced to conceal their abortions and seek care from providers outside of the camp or use unsafe or unapproved procedures like salt or Flagyl [16]. Young, vulnerable girls and women are more likely to undergo these kinds of unsafe abortions and abortion techniques, particularly in countries where abortion is prohibited (like Nigeria) or severely regulated [16].

Relevant governmental and non-governmental organizations have shifted their efforts to make it easier for internally displaced people to go back home to places where security has since improved [18]. Collectively, they assisted citizens in overcoming the various difficulties brought on by relocation [18]. However, despite the growing focus on improving health outcomes for this population, they still face limited access to quality SRH treatment [19, 20]. Poverty, sociocultural disparities, language and literacy issues, and other factors may hinder access to SRH services like contraception and sexually transmitted infections (STI) prevention and treatment [7]. Populations that have been internally displaced are more vulnerable in terms of their sexual and reproductive health, particularly women and girls. This includes a rise in the prevalence of unsafe abortions, high maternal mortality, and sexual assault, especially in the Nigerian context. The results of this study could be very important in developing more efficient health interventions and policies that are adapted to the requirements of displaced women, enhancing their general health and quality of life [16, 21].

Internally displaced people are usually forced to flee or leave their homes due to conflict or other factors, which subject them to a high level of suffering. Studies around the IDP situations in Nigeria have less focused sexual and reproductive health aspects of these vulnerable populations. Attention has been majorly concentrated on the physical structure of the camps, camp management, and health resilience practice [22, 23]. The same pattern of interest was observed in Mali [24], Iraq [25] and other high-income countries [26]. Also, risk factors for sexually transmitted infection were examined in the Abuja camp among women of reproductive ages [27, 28] with a focus on women and adolescents 10–19 years. [29] audited the healthcare provision in the IDP camps in Nigeria and found a higher likelihood of water, food, and air-related disease and poor quality of life among the IDPs. Studies are sparse on the SRH needs, barriers, and coping strategies of these vulnerable populations. This study, therefore, aims to explore the sexual and reproductive health needs, barriers, and coping strategies of internally displaced women of reproductive ages in north-central Nigeria.

## Methodology

### Study design

An exploratory design using qualitative methods was adopted to explore the SRH needs, barriers and coping strategies of selected women of reproductive age in the selected IDP camps in Abuja and Benue on the dates 08/01/2024 to 28/02/2024. This approach allows for a better understanding of the study from a naturalistic perspective and gives a straight description [21, 30]. In using this method, researchers acknowledged and prioritized the voices and perspectives of participants, providing a platform for marginalized or underrepresented groups to share their experiences and viewpoints. This presents information from the view of women of reproductive ages in the IDP camps using In-depth interviews.

### Research setting

Nigeria, located in West Africa between longitude 3°E and 15°E and latitude 4°N and 14°N, is the most populous country on the African continent, boasting a population exceeding 206 million. Spanning a land area of 923,768 km^2^, Nigeria is organized into 36 states and a federal capital, distributed across 6 geopolitical zones: North-west, North-east, North-central, Southeast, South-west, and South-south, encompassing a total of 774 Local Government Areas (LGAs). Within Nigeria, approximately 53% of women of reproductive age reside in IDP camps, comprising about 1.73 million individuals of childbearing age [31]. This study was conducted in Abuja and Benue State, specifically chosen due to their significant concentration of functioning IDP camps in Northern Nigeria. Also, at least five distinct IDP camps within Abuja collectively house 13,481 individuals. Noteworthy, these camps include Lugbe IDP Camp, Area One IDP Camp, New Kuchingoro IDP Camp, Malaysian Garden and Kuje IDP Camp. Despite the challenging conditions within these camps, the inhabitants still perceive them as relatively comfortable places to reside.

Also, numerous individuals from Benue State, both locals and outsiders, were displaced by violent encounters with herdsmen in their communities in the past. Presently, they find shelter in specified camps throughout the state such as in Guma local government where we have Ortese and Daudu Camps, and also in Markurdi local government where we have another camp at the North Bank area. The deplorable living conditions they endure, coupled with the sense of being forsaken by the authorities, could bring tears to the eyes of anyone who visits these camps. Inhabiting these internally displaced persons (IDP) facilities is essentially akin to inviting a tragic fate. The study sites in Abuja are Area One Camp and Malaysian Garden Camp, and in Benue, we visited Ortese and Daudu Camps.

### Study population

The study population for this research consists of Internally Displaced Persons residing in (Area One and Malaysian Camps) Abuja and (Ortese and Daudu Camps) Benue State camps, Nigeria, with a specific focus on women of reproductive age (15–49 years). This diverse group of participants is essential for a comprehensive understanding of the SRH needs, challenges and coping strategies. In Nigeria, there are 53% of women of reproductive age in the IDP camps. In Abuja camp, there are 11,155 women of reproductive age in the camp [27]. In Benue camp, there are more women than men [32].Study population and recruitment.

The participants selected for this study were women of reproductive ages between the age of 20–49 years, who were willing to participate in the study and did not need parental consent. The method was used because the research needed women with a wealth of experience in discussing sexual needs and those who would not feel pressured or exposed while discussing their issues. These women were purposefully recruited using locally accepted methods of establishing contact [33]. The researcher connected with IDP camp guides or gatekeepers, who facilitated introductions to camp officials. During these meetings, the researcher clarified the study objectives and obtained consent to conduct research involving women aged 20–49 residing in the IDP camps. The target participants were women in this age group, recognized to encounter sexual and reproductive health (SRH) challenges. Camp officials collaborated with the research team to locate specific areas within the camps where these participants could be found. They also assisted in scheduling meetings and facilitating communication with women participating in individual discussions which focused on SRH needs, challenges and coping strategies. For the IDIs, 14 women of reproductive-aged 20–49 years participated in the in-depth conversations.

The inclusion criteria were only women of reproductive age (20–49 years) and residence in the selected IDP camps.

The exclusion criteria comprised participants who were very ill and were not residing in any of the IDP camps. Overall, 14 female participants were recruited in this study.

### Pretest

The research assistants, already knowledgeable and acquainted with the study, underwent customized training focused on fundamental qualitative research methods and a tool review. A preliminary tool assessment was conducted, followed by seeking feedback from research participants on question clarity, which they affirmed. Consequently, the researcher adopted the interview guide with minimal modifications.

### Data collection

The trained Research Assistants (RAs) who helped to collect the data were both males and females between the ages of 25–49 years. They were identified from a pool of experienced persons from the district and in the states where the study was conducted. Some of the RAs were graduates and were able to speak both English and the local languages, best understood by most of the women in the IDP camps. Data were collected using translated and pre-tested in-depth interview guides that were developed based on previous studies and reviewed about SRH issues [34]. The interviews were recorded verbatim, transcribed, and translated back to English.

### Ethical considerations

Ethical consideration was sought from The Federal Ministry of Health Abuja, Nigeria, the Federal Capital Territory Health Research Ethics Committee with FHREC approval number (**FHREC/2023/01/231/13-11-23**) and, the National Health Research Ethics Committee of Nigeria with HREC assigned number (**NHREC/01/01/2007-30/10/2023).** Informed verbal consent was also obtained from all participants in the selected IDP camps and the verbal consent was noted and the study recruited only those who consented. Anonymity and confidentiality were ensured during data collection and also ensured for storage, and reporting.

### Data analysis

Audio recordings from the IDIs underwent labelling, verbatim transcription, and English translation by experienced research assistants fluent in the respective languages. The recordings were stored securely. Deductive, theme-based coding was conducted, resulting in the development of a codebook. Transcripts were then entered into NVivo version 11 for data coding. The data underwent content analysis. To ensure reliability, coding was overseen by a research expert under the guidance of the Principal Investigator. The expert reviewed the transcripts, discussed emerging issues, and identified common themes. Additionally, two transcripts were selected and thoroughly analyzed, with each response interpreted uniquely, following the procedure outlined by Braun and Clarke to develop a comprehensive codebook [35, 36]. The themes from the discussions and results that contribute to understanding of the study findings are presented below to complement the survey results. Similarly, themes generated sun-categories which gave a more general description of the contents.

## Results

### Socio-demographic of the participants

A total of 14 women participated in this study (see Table 1 below). Five participants were between ages 35–39 years, three participants each were between ages 40–44 years and 45–49 years respectively, two participants were between ages 25–29 years and only one was between ages 30–34 years. Of the participants interviewed, the majority (64.3%) practised Christianity while the remaining 35.7% practised Islamic religion. However, most of the women (35.7%) were from the Malaysia camp, 28.6% were from the Ortese camp, 21.4% were from Dauda to 14.3% were from Area One Durunmi Camp.

**Table 1:** Socio-demographic characteristics of the participants.

Variables		Number (%)
**Data Collection Method**		**IDIs**
**Total number**		**14 (100)**
**Gender**		
Females only		14 (100)
**Age**		
**Age range**	25–29	2 (14.3)
	30–34	1 (7.1)
	35–39	5 (35.7)
	40–44	3 (21.4)
	45–49	3 (21.4)
**State**	Abuja	7 (50.0)
	Benue	7 (50.0)
**Religion**	Islamic	5 (35.7)
	Christianity	9 (64.3)
**IDP Camps**	Malaysia (Abuja)	5 (35.7)
	Durunmi Area One (Abuja)	2 (14.3)
	Ortese (Benue)	4 (28.6)
	Dauda (Benue)	3 (21.4)

## Sexual and Reproductive Health (SRH) Needs of Women in the IDP Camps

### Family planning needs

#### Spousal sensitization on the importance of family planning

The family planning needs of women were mentioned in the study, the women reported that they know more about family planning and method options but they are unable to use it because of their spouses. Our participants mentioned the difficulty they face in making their husbands realize the need for contraceptive uptake and that their decision to use was never supported by their husbands. Despite the report that some of the women in the camp have no less than 15 children, many of the women know the importance of family planning in child spacing and limiting but the husbands were not in support Hence, the barrier to contraceptive adoption among the women.

*On FP they are getting to understand the importance and gradually accepting it because the number of those accepting it is increasing gradually. And there was a seminar for their husbands a few times so they allowed them to accept it.* ***(27 years old Muslim, Durunmi Camp Abuj***a)

*Women here need so many things and thank God some are getting to understand the importance of FP because before one woman could have as many as 8 to 10 to 15 children but they are now realizing the fact that it is not about having children*, *but the husband need quality training* ***(27 years old Muslim Durunmi Camp Abuja***)

*We need enlightenment for our husband to allow FP **(27 years old Muslim Malaysia Camp Abuja)***

*On Family planning we need more counselling because some want but are scared of their husband*, *so if the husband Knows the importance to women will take it me i have 5 children already and I do not want to add to them because the ones I have self are not getting all the care they need*. ***(49 years old Muslim Malaysia Camp Abuja***)

#### Planning methods needs

Family planning itself is what the women claim they need. The women report the efforts of health workers in creating awareness in the camp, however, despite this effort, the methods are not readily available for use. The women stated that any time they visit the hospital they always tell them that the method choices are not available, hence the need to revisit the facility. So, the women mentioned the need for the government to provide family planning method choices for them to use. Specifically, some women mentioned the need for emergency contraception to prevent pregnancy, and some also mentioned difficulty in getting condoms to prevent unwanted pregnancy among the women.

*On family planning, they conduct awareness through some health officials in the camp, telling us the benefits of family planning, and some methods they show that we can use to control pregnancy and childbirth, like condoms, injectables, and implants. But the drugs are not always available, we go there they will tell us to come tomorrow and so. So, we need family planning to control childbirth and pregnancy that is not planned for****. (35 years old Christian, Ortese Camp Benue***)

*Personally*, *for me and even for many women here*, *we need family planning services especially the use of emergency contraceptives*. ***(35 years old Christian*, *Daudu Camp Benue***)

*They show us some methods like long-term and short-term methods but even condoms are difficult to get here*. *So these services should be made available to control unwanted pregnancy* ***(35 years Christian*, *Daudu Camp Benue)*.**

### Antenatal need for women in the internally displaced camp

#### Maternity clinic facility for IDP women

A maternity clinic is one of the needs of the women in the internally displaced camp. They mentioned that the women usually go out of place and pocket to access antenatal services. They explained that the primary health centre where they deliver is far from them and that it would be a lot easier if they could situate a facility for delivery in the IDP camp for these women to access easily. As elaborated by a respondent, it was said that women do go through labour at night and have no car to transport such a woman to the nearby clinic, building a health facility for delivery will go a long way to improve health outcomes and also increase sexual and reproductive health services uptake. This need cuts across both locations visited for the study.

*In terms of delivery of a child, they have the facilities for that and women are directed to go to the primary health care centre along Ukpian road or hospitals in town which they are asked to pay money for if this is looked into, it will help a lot, we are displaced and we don’t have sources of livelihood.* ***(35 years Old Christian, Daudu camp Benue***)

*On Antenatal and Postnatal we need a hospital in the clinic because we don’t have any and some women will labour at night going to Waru Hospital is far*, *the body is weak and the husband doesn’t have a car so we need a hospital and even for every body’s health needs*. ***(27 years old Muslim*, *Malaysia camp*, *Abuja***)

*On Antenatal and Postnatal care services*, *we need consistent attention for successful delivery and Postnatal care*, *though the woman leader and TBA here is seriously trying and it has been successful but more medical personnel too will be appreciated*. ***(27 years old*, *Muslim Durumi Camp*, *Abuja***)

#### Adequate medication for women in need

According to the report received from the women in the camps visited, they mentioned that the health workers are trying their best to provide for their sexual and reproductive health needs, however, they still need medications, such as drugs for pregnant women, and pills for protection during sex among others. All these the women claimed are not available in the health facilities visited.

*Same with antenatal and post-natal, they currently provide the services but the drugs are not currently available and sometimes they ask the women to buy it outside and they don’t have money to buy it outside.* ***(35 years old Christian, Daudu camp, Benue***)

*On Antenatal and Postnatal care services*, *we need if not permanent visiting health workers to come and give them care and counselling and drugs are needed here* ***(45 years Muslim*, *Malaysia Camp*, *Abuja***)

### Sexual transmitted infection and HIV needs of women in the camp

#### Need for modern toilet facility

Women in this study were asked about their needs regarding sexually transmitted Infection and HIV/AIDs in the study locations, they explained that in the case of sexually transmitted Infection (STI), there is a need for the government to provide a modern toilet for them, they reported that many of them contract infections because they use a common toilet shared by many. They feel what they contracted is sexually transmitted infections through the toilet though what they contracted was toilet infections. Given this, they requested for modern toilet as they don’t have any in the camp where they stay.

*On STI it is very plenty because we don’t have toilets so we are praying for the person that will support us with it* ***(49 years Muslim, Malaysia Camp***)

*On STIs*, *it is very common because we don’t have toilets and women easily get infections even from their husbands even some of our husbands have two or three wives so we don’t know how the infection is spreading*, *but it is a big challenge for us* ***(47years Muslim*, *Malaysia Camp***)

*We keep having reinfection because we don’t have toilets*. ***(43years Muslim*, *Durunmi Camp***)

#### Adequate testing KITs and treatment for STI and HIV

The women of reproductive age interviewed reported the need for adequate treatment and testing for HIV and sexually transmitted infections in the camps visited. This is because many of them are infected and there are no drugs or any forms of treatment available for treatment and testing. They reported their concerns over reinfection due to the lack of testing apparatus and kits, they expressed their worries about the need for the government to provide them with kits and treat the infected person to avoid reinfections in the camp. This opinion cut across most of the locations visited for the study. They also reported that the only people the health workers care about when it comes to HIV and STIs are pregnant women and those who are raped. Others are being neglected, hence the need for adequate treatment and toolkits for testing in the camps.

*My basic SRH needs is quality treatment for Infection am a widow and I have grown up with girls I understand how disturbing infection is and the available drugs in the clinics are not enough and their effectiveness is not strong.* ***(43years Muslim, Durunmi Camp, Abuja)***

*In this camp, what we need is good treatment for infection because it affects plenty of us. **(36 years Muslim, Malaysia Camp, Abuja)***

*We have a challenge with HIV testing we come they tell us they don’t do tests here, but when it’s a pregnant woman or rape case they treat and test the person and they counsel **(35 years Christian, Daudu Camp, Benue)***

*STI*, *they treat but only pregnant and rape cases they treat*, ***(35 years Christian*, *Ortese Camp*, *Benue***)

### Gender base violence and rape cases needs of women in the camps

#### Care and counselling center for gender-based violence and rape

As mentioned by the women in the camp, they explained that when a rape situation occurs, it is usually difficult to reach the health worker for treatment or care. They reported that most of the incidence happens at night and they always need to wait till the next day before they report the case, they sometimes have to call the hospital to tell them the severity of the incident. With that, they still have to wait till the next day to provide care for the victim. They say it is usually dangerous for such victims, hence the need for GBV and Rape care and counselling centre for the victims to access immediate and first aid care.

*On rape and gender-based violence, they treat patients but if it happened during the weekend, we are to wait till they come on Monday or the camp official put a call before they come and treat the victims. They treat here initially but now, it’s pregnant women and cases of rape when they handle they treat or they refer you to Ortese IDP camp you spend money that you don’t have for transport.* ***(35 years Christian, Ortese Camp, Benue***)

*In cases of rape*, *we don’t have the means to fend for ourselves*, *we just wait for the medical officers to come or we put a call and inform them how severe the situation is and they send someone to come and handle it*. ***(42 years Christian*, *Daudu Camp*, *Benue)***

#### Relationship counselling for boys and girls in the camp

The women in the camp emphasized the importance of counselling in the case of rape and gender-based violence. They opined that when a girl child is counselled on when and how to manage their lives, who to have relationships with and the boys are adequately trained from home they will grow to be responsible people in the community, hence the need for counselling to avoid rape and gender-based violence.

*On rape occurrence and gender-based violence, we need continuous advice for our girls and boys though this place is good they should be guided.* ***(43 years Muslim, Durunmi Camp, Benu***e)

#### Coping strategies for sexual and reproductive health needs of women in utilizing SRH services in the camp selected

This study also explored the coping strategies of women’s sexual and reproductive health needs in the camp. According to the questions asked, we asked the women how they cope in case of absence or insufficient sexual and reproductive health services in the camp. The women reported how they cope based on each of their sexual and reproductive health needs in the camp. They reported coping strategies in the absence of insufficient family planning methods, they reported how they cope when there are no hospitals to run antenatal clinics and other postnatal services. While they contract sexually transmitted infections, they told us the alternative method. Detailed coping strategies were also discussed based on each sexual and reproductive health needs.

### Family planning coping strategies among women in the camp

#### Prayer during unprotected sex as a coping strategy

According to what we recorded from the women, it was mentioned that because of a lack of family planning methods options and no money to buy condoms, the women reported having unprotected sex and praying they wouldn’t get pregnant. So, in a way, prayer is a coping strategy they resolve to protect themselves from unwanted pregnancies.

*It becomes a problem o, hmm, we sometimes in family planning when we don’t have access to the methods, that I am using like condoms, hmm me and my husband just do it like that and pray I don’t get pregnant.* ***(35 years old, Christian, Ortese camp, Benue***)

*It becomes a problem o*, *some of us who use condoms*, *when it’s not available*, *I meet my husband like that*. ***(35 years old*, *Christian*, *Daudu camp*, *Benu***e)

*We try our best to abstain but when your husband needs you and the family planning methods are not available*, *is to have a problem with your husband you just do and wait for the outcome* ***(40 years old*, *Christian*, *Daudu camp*, *Benue***)

*We are not bothered about FP*, *we do it like that and pray* ***(36 years old Muslim*, *Malaysia Camp*, *Abuja***)

#### Use of waist medicinal beads as a coping strategy

Waist beads are one of the ways women prevent pregnancy when there are no modern family planning services. They reported that most of the time they use waist beads to prevent pregnancy, this is considered a traditional method of prevention and they reported that it is very effective in preventing pregnancies.

*coping strategies on family planning is some are using the local way of putting medicinal beads on their waist* ***(27 years old Muslim, Malaysia Camp Abuja***)

*Our mothers and grandmothers use the natural method*, *so we also use it and it’s ok* ***(43 years Old Muslim*, *Durunmi Camp*, *Abuja***)

### Women’s coping strategies for antenatal and post-natal services utilization

#### Traditional herbs and trees to reduce pain during and after delivery

The women reported that when there are no drugs to use during pregnancy or the postnatal period, and there is no sufficient money to purchase the drugs they need, for the mother and child to be in good health and reduce pain during delivery and postnatal pain, they resolved into herbs and trees. This is considered traditional medicine as a coping strategy used to keep fit and have a safe delivery.

*On antenatal and post-natal services, when a woman complains of stomach pain and the clinic doesn’t have drugs available, we use the traditional methods.* ***(40-year-old Christian, Daudu Camp. Benue***)

*for women pregnant and nursing mothers*, *when drugs are not available*, *they wait for but when it takes too long and they don’t have money to buy it*, *they resort to herbal medicine*. ***(35 years old Christian*, *Ortese Camp Daudu***)

#### Tradition birth attendants as coping strategies for antenatal services utilization

Women also reported that when there are no drugs or any form of attention or access to reach the hospital in other to utilize health care services, they always resort to the use of traditional birth attendants for delivery and antenatal care.

*On Antenatal some still depend on the information they get from home; some have TBA at Kabusa they go to for Antenatal care****. (27 years Muslim, Malaysia Camp, Abuja***)

*On Antenatal the elderly mothers among them used to guide and counsel them so they become more comfortable giving birth at home and might not go for post-natal care services at the facility*. ***(36-year Muslim*, *Malaysia Camp*, *Abuja***)

#### Barriers to utilization of sexual and reproductive health services in the camps

The utilisation of sexual and reproductive health services is hampered by several factors, as reported by the participants. Women sometimes have a positive attitude towards the use of SRH services but cannot use them because of one problem or another. Below are some of the mentioned barriers to the utilisation of SRH services among internally displaced women. This barrier will therefore be discussed based on each of the SRH services measured in this study, which are family planning, antenatal and postnatal care, STI and HIV services, and rape and gender-based violence services.

#### Barriers to family planning services utilization

The use of family planning is highly important in reducing unwanted pregnancies and improving the health outcomes of women. The women in the camps visited also recognised its importance; however, despite their desire and willingness to use it, they encountered some barriers in the process. Below are some of the barriers encountered while accessing family planning services in the internally displaced camp.

#### Unavailable family planning methods

As reported by the women, they mentioned that they understand the importance of family planning, and many of them are willing to use it, but anytime they visit the hospitals, they will counsel them and select the appropriate method for them; unfortunately, the method options are never available. Some even mentioned that common condoms cannot be afforded by many of them and that, with the help of the doctors without bother, they usually come to camps to distribute condoms but because of the increase in the population of the people in the camp, condoms are not always sufficient. This has become a major barrier to the utilisation of family planning services at the camps visited. Below are some of the excerpts as reported:

*The barrier to FP is that it is not always available for us in the camp. For instance, since I got into this camp, I don’t know many places, even the closest hospital.* ***(27 years, Muslim, Durunmi Camp Abuja***)

*The barrier to FP is that it is not always available for us in the camp* ***(27-year-old Muslim*, *Malaysia Camp Abuja***)**.**

*These people (MSE) doctors without borders bring drugs and those materials but the population is growing*, *and the people are much here*. *If they bring condoms to share*, *it is not enough*, *and like that*. ***(25-year-old Christian*, *Ortese Camp*, *Benue***)

*Of course*, *we have challenges in the aspect of family planning; they will come but sometimes these methods are not available*, *like condoms and others*. *(Injectables and implants)*. *They tell us to go and come back when we do and the options are not available*. *Women get pregnant and it’s not usually planned*. *That’s when you look; you see a lot of pregnant women in the camp***. *(35 years Christian*, *Daudu Camp*, *Benue)***

#### Distance to health facility

One of the barriers mentioned by women to utilising family planning services is the distance from the camp to the health facility where they will get the method of their choice. They mentioned that they don’t have transportation to go to the clinic, preventing them from going to the health facility and getting the family planning method needed.

*distance to the hospital in Waru is far, to access family planning will cost much transport* ***(27 years Muslim, Malaysia Camp, Abuja***)

*distance to the hospital in Garki is not too far according to those that have been there but no transport fare to go there*, ***(27 years Muslim*, *Durunmi Camp*, *Abuja***)

*we can’t go to the clinic at any time to get condoms and some of us use condoms*. ***(35 years*, *Christian*, *Daudu Camp*, *Benue***)

#### Low acceptance rate of family planning methods

According to the women, they reported that one of the barriers to the utilization of family planning services is the low acceptance rate of family planning services. They mentioned that some still don’t have the understanding and thereby do not support the use. They mentioned the need for the health workers to constantly visit the camp to encourage and educate them to use it.

*some are scared but if they are coming regularly gradually the women will be accepting it. And some are still not in support of it rather anything that comes their way they are okay with it.* ***(49 years Muslim, Malaysia Camp, Abuja***)

*low acceptance due to lack of education on it*. ***(27 years Muslim*, *Malaysia Camp*, *Abuja***)

#### Barriers to utilization of antenatal and postnatal care services

Our study explored reasons women are not utilising antenatal and postnatal care services for positive health outcomes. The women opined that there are adequate health facilities for delivery, though it is outside the camp, and women are not willing to deliver there. The reasons are discussed below:

#### Lack of finance to access antenatal/postnatal services

As mentioned by the women, the main reason why women do not patronise the available health facilities for delivery is because of a lack of finances. They explained that most of the time when they visit the nearby primary health center for delivery, they are always referred to another facility due to a lack of resources and drugs to use. Due to this, women are made to pay a huge amount of money to access care in the referred hospital; this is what many of the women cannot afford, thereby preferring to use traditional birth attendance or delivery at their camp of residence. So, lack of finance is the major barrier to antenatal care services in all the camps visited.

*The barrier we have, basically, is finance. Going to the clinic to buy things for the baby to even eat is a big problem because many of our husbands don’t have jobs and are just managing their lives.* ***(49-year-old Muslim, Malaysia Camp, Abuja***)

*Antenatal and postnatal*: *as said*, *they take care of the pregnant women and see them after delivery*, *but they don’t take delivery here; they tell us to go to another hospital; it is challenging for the women; and when we can’t afford the charges at the hospital*, *they end up giving birth in the camp before bringing the child for postnatal service*. ***(35-year-old Christian*, *Ortese Camp*, *Benue***)

*In antenatal and postnatal care*, *they provide what they need at some point but the drugs are not always available and sometimes they ask the pregnant women to buy them outside*. *And they don’t take delivery here; when the women are due*, *they are directed to a primary health care centre for delivery*, *where they ask them to pay some money*. ***(40 years Christian*, *Daudu Camp*, *Benue***)

*The barriers to antenatal and postnatal care are distance and finance* ***(36-year-old Muslim*, *Malaysia Camp*, *Abuja***)**.**

#### Personal beliefs affect utilization of antenatal and postnatal services

The women in the camp also mentioned that people’s way of thinking and their personal beliefs sometimes interact with their reproductive health decisions. They explained that some women have nonchalant attitudes towards going to the hospital for delivery; they believe it’s a waste of time and resources, while others believe in the efficiency of traditional birth attendants for delivery. All these, in a way, serve as barriers to the utilisation of antenatal and postnatal services by women in internally displaced camps.

*On the antenatal and postnatal care services barrier, there is some belief that going to the hospital for checks and delivery is a waste of time.* ***(43-year-old Muslim, Durunmi Camp, Abuja***)

*On antenatal and postnatal care services*, *some still believe in the traditional birth method so they believe that since they are not sick*, *they don’t need a hospital as their grandparents didn’t go to the hospital*. ***(27-year-old Muslim*, *Malaysia Camp*, *Abuja***)

#### Barriers to the utilization of sexually transmitted infections and HIV services

Utilisation of sexually transmitted infections and HIV services is very important in improving the reproductive health of women in the camp. However, women mentioned some barriers hindering their efforts to utilise the services. This will be discussed below.

#### Selective treatment

The women in the camp mentioned that the health workers only focus on the treatment of emergencies and critical cases of STIs and that this treatment is also age-focused. They said that the nurses won’t treat you if you are not pregnant or if you are over the age of 25. This is said to affect the quality of treatment the women should have access to in the camp.

*On HIV and STI treatment, initially, they had facilities for tests, but they have gone now. If one infection they don’t treat, only pregnant women, when you are a woman of 25 years, they don’t attend to you here; they ask you to go to the camp centre. The moment you tell them you are over 25 years old, they don’t treat you. The centre treats those under the age of 24 and below.* ***(42 years Christian, Daudu Camp, Benue***)

*On STIs*, *they treat infections but they don’t test for HIV here*, *only for pregnant women and cases of rape*. ***(40 years*, *Christian*, *Daudu Camp*, *Benue***)

#### Barriers to gender-based violence and rape reproductive health services utilization

Though gender-based violence and rape cases were not commonly reported in the camp and those who experienced them mentioned they happened outside the camp, to them, the camp is somehow safe. However, for the few who acknowledge the occurrence of GBV and rape, they mentioned why people do not utilise the services provided or available for these occurrences and below are the points mentioned:

#### Fear of stigmatization

The only significant point mentioned to affect the utilisation of the services available for rape cases and victims of gender-based violence is the fear of stigmatization. Fear of stigmatisation affects the reporting of rape cases and GBV cases. They explained that the way society views the victims and behaves towards them discourages them from reporting and talking more about accessing the services available.

*In cases of rape and gender-based violence, the victims are ashamed to come and speak, though the MSF staff do encourage us but when people find out that you were raped, they look at you differently and some would say you just made the story to cover your infidelity.* ***(35-year-old Christian, Daudu Camp, Benue***)

## Discussion

Over 89 million people had been forcibly relocated worldwide by the end of 2021, with the majority of them being internally displaced people (IDPs) and refugees. Sub-Saharan Africa is linked to one-fifth of the world’s refugees. Approximately 2.2 million people, mostly women and children, are being displaced in northeastern Nigeria as a result of the Boko Haram insurgency [19]. This highlights a significant disruption to the availability/barriers to sexual and reproductive health services and coping strategies of internally displaced women of reproductive age. The study explored the sexual and reproductive health needs, barriers and coping strategies of women of reproductive ages in North Central Nigeria. The major sexual and reproductive health needs of the women living in the IDP camps are emergency contraception, condoms, spousal sensitization, provision of maternity clinic facility, modern toilet facility, adequate STI testing kits, and relationship counselling for the boys and girls in the camps. With lack of the lack of these basic things, the participants reported the use of prayer, medicinal waist beads/herbs, and traditional birth attendants as coping strategies. Also, the women were said not to utilize the available SRH services at the health facilities because of preference for family planning methods, proximity to the health facilities, lack of finance, personal beliefs and fear of stigmatization.

### Sexual and reproductive health needs of women in the IDP camps

Women of reproductive age, especially the younger age group should have access to basic SRH services, as it has been reported that the younger age group due to puberty age tend to be sexually active and engage in risky sexual behaviour [37, 38]. The study participants mentioned each of their needs based on the outcome variable of the study which sexual and reproductive health needs mentioned in the methodology section. Each of the SRH needs will be discussed one after the other for a comprehensive understanding.

As regards family planning, the participants reported that they have a comprehensive understanding of family planning services, but they are limited by the ignorance of their spouses. Several studies have shown the influence of spouse (husband) in decision-making in northern Nigeria, as they have the belief in male dominance in all spheres of life, including decisions on the adoption and utilization of sexual and reproductive health services by their wives [39, 40]. Male knowledge, whether educated or not, or whether they are aware of the importance of family planning is imperative for the utilization of family planning in northern Nigeria [41]. Husbands’ poor perception of the context of family planning may be one of the reasons why they do not allow their wives to utilize family planning [42]. Despite that some of the women had a lot of children, many of the women were reported to know the importance of family planning in child spacing and limiting but could not convert the knowledge into practice because of the lack of support from their husbands. Therefore, the women clamour that the husbands should be enlightened on the importance of family planning, so their perception of sexual and reproductive services can change.

Awareness and knowing the benefits of health services contribute greatly to their utilization. Although the healthcare workers created awareness and taught about the benefits of family planning by rallying around the camp the family planning choices of the women in the IDP camps were not readily available [43]. Family planning methods like oral contraception and barrier methods (condoms). These choices might be because they are the easiest to use without their husband getting to know (emergency contraception) or the fact that they believe their husbands’ belief that condom usage will not cause any physiological changes in the woman [44].

Maternity clinic facility which is one of the basic health needs of women of reproductive age wasn’t available in some of the IDP camps in this study, which in turn contributes to the delay in achieving SDG goals [45]. One of the causes of maternal death is complications during pregnancy or at the time of birth [46, 47], this can be reduced substantially if maternity clinic facilities are made available and accessible to pregnant women and women of reproductive age [43]. Integrating antenatal care into healthcare services provided in the IDP camps is essential to help reduce the surge of SRH services deficiency in IDP camps in Nigeria. As elaborated by one of the study participants, there’s difficulty in transporting labouring women to health facilities for delivery. Building a health facility for delivery will help improve health outcomes and uptake of SRH services.

As reported from the horses’ mouths in this study, many of them are infected with STIs/HIV and there are no treatments or drugs available. The focus of HIV testing and treatment is mainly on pregnant women while others were neglected. If other women are neglected and not treated, even those pregnant women who were treated are at risk of reinfection due to the one-to-many wife (polygamous family) common in northern Nigeria [48, 49], recalling that the major route of HIV transmission is through sexual intercourse [50]. Internally displaced women are vulnerable to gender-based violence. Hence, there should be gender-based violence care and counselling services available to IDP women. The study revealed that IDP women don’t get an immediate intervention as most of the incidents happen at night. This means that proximity to GBV care is far which could be dangerous for the victims.

#### Coping strategies for sexual and reproductive health needs of women in utilizing SRH services in the camp selected

The study participants accrued some coping strategies for the unavailability or insufficient sexual and reproductive health needs in the camp. Africa, Nigeria especially has been reported to be a religious country, where Prayer is believed to solve everything [51]. The participants reported they engage in prayers hoping it would protect them from unwanted pregnancies. Women of reproductive age before orthodox medicine use some traditional family planning like counting the days of the month, herbs, and traditional waist beads [52]. The study participants use waist medicinal beads as a coping strategy to prevent pregnancy and this traditional method has been proven to work overtime. However, these coping strategies highlighted only prevent unwanted pregnancy but weren’t reported to treat/cure HIV and other STIs. Hence, the women are prone and at risk of contracting sexually transmitted infections.

On the side of the healthcare workers, they highlighted that usage of traditional herbs and trees are used to reduce pain during and after delivery. These herbs serve as local anaesthesia and analgesics [53]. Studies have shown that pregnant women should stay away from herbs until it has been vetted for safety [54]. Women who had no access to or could not afford sexual reproductive services utilize traditional birth attendants for delivery and antenatal care. This could expose mothers and neonates to maternal/neonatal infections [55].

#### Barriers to utilization of sexual and reproductive health services in the camps

One of the major barriers highlighted by the women is the unavailability of a family planning method of choice after counsel from the healthcare workers. Unavailability and lack of access to family planning reduce utilization rate. Another factor that can reduce the utilization rate of family planning is the proximity of health facilities that offer family planning services. The study participants reported that far distance and lack of finances to transport themselves to the health facility prevented them from using family planning. Adequate awareness and knowledge of family planning are contributors to the acceptance of family planning. Lack of finance made antenatal/postnatal care inaccessible because of the service charge on them. Women living in IDPs may not have the capacity to pay for these charges and may prefer low/no-cost antenatal/post-natal services [56]. Some of the respondents also have more trust in traditional birth attendants compared to healthcare workers which may be due to their low knowledge of modern contraception and probably the relationship between the healthcare workers and the women. The quality of treatment the women should have access to in the camp has been affected by age and pregnancy status stereotyping. Women who were younger than 25 years or pregnant had a higher priority for STI treatment compared to others.

Internally displaced women are vulnerable to gender-based violence. Hence, there should be provision of gender-based violence care services. Although rape was reported not to be common in the camps for the few that reported its occurrence, fear of stigmatization was a major barrier to utilizing the available service. Stigmatization and discrimination are major barriers to rape case reports, female gender mutilation and STI treatment service utilization [57].

## Conclusion

It is safe to conclude that, sexual and reproductive health services especially family planning and Postnatal services were not available for the women at the IDP camps. This study established that selective STI treatment is a major barrier to the utilization of SRH services. In the absence of sexual and reproductive health services, women living in the IDP camps engaged in traditional contraception for the prevention of pregnancy but were still susceptible to STIs.

## Recommendations

The government should build more health infrastructures in the IDP camps and integrate sexual and reproductive health services into the existing facilities. Health education should be done among males/husbands in the IDP camps to ensure they understand the context of contraception to clear their doubts. Similarly, health education should be done among women to ensure that they are knowledgeable about the impacts of sexual risk behaviours so they can desist. Also, there should be adequate provision for family planning methods to help meet the choices made by the women in the IDP camps. Lastly, traditional birth attendants should be trained in basic care and hygienic conditions while delivering babies to prevent maternal and neonatal infection.

## Strengths and limitations of the study

One of the strengths of this study is that it employed a primary method of data collection which affords a detailed and inclusive curation of data from the internally displaced women and gives a feel of the state of things in the camps. Primary data offers more up-to-date information about a population compared to the secondary data, hence, a level of timeliness of the report of this study. More so, the study was able to give an overview of the state of women in IDP camps in the North central part of Nigeria, a case which has not been in the past years. This study shed more light on the needs, barriers and coping strategies of sexual and reproductive health of women in the IDP camps in the North Central region of Nigeria. One major limitation encountered is the movement of the internally displaced women in search of food and means of subsistence, IDP camps visited most especially Benue state camp harbours farmers and many of the women had to go to farm to feed their families, so we had to reschedule some of the data collections process till they were available. This elongated data collection days, processes and spending. However, the challenges were mitigated by waiting and making sure we sampled adequate and eligible respondents at all costs.
